# Two-stage exchange for PJI with co-existing cerclages for fracture: higher rates of early re-infections and difficult to treat microbes

**DOI:** 10.1007/s00402-022-04361-0

**Published:** 2022-01-28

**Authors:** Daniel Karczewski, Maximilian Müllner, Christian Hipfl, Carsten Perka, Michael Müller

**Affiliations:** Center for Musculoskeletal Surgery, Department of Orthopaedic Surgery, Charité- University Medicine, Charitéplatz 1, 10117 Berlin, Germany

**Keywords:** Periprosthetic joint infection, Fracture, Cerclage, Two-stage exchange

## Abstract

**Introduction:**

Periprosthetic joint infections (PJI) with osteosynthesis material for contemporaneous fractures are a challenging, yet poorly described condition. This study will analyze PJI with co-existing fractures treated with cerclages and two-stage exchange.

**Materials and methods:**

Patients with and without cerclages for coexisting periprosthetic fractures, undergoing two-stage exchange for PJI of hip or knee, between 06/2013 and 02/2016, were compared concerning baseline characteristics and re-infection rate in the course of a 2 year follow-up. All patients were treated with a standardized two-stage protocol. A PJI was defined according to the EBJIS criteria. All foreign material, including cerclages, was sent in for sonication for microbiological analysis.

**Results:**

Ninety-six patients treated with two-stage exchange for PJI could be included. Co-existing fractures treated with cerclage were identified in nine patients (9.3%, study group). Diaphyseal femoral simple in five cases (AO2A3) and proximal intertrochanteric in three cases (AO1A3) were the leading fracture locations. In one patient, cerclage implantation was performed prior to prosthesis explantation, in six, during prosthesis explantation, and in two, in the course of prosthesis reimplantation. The study group showed a significantly higher rate of difficult to treat microbes (44.4%; 8.0%; *p *= .001), Charlson Comorbidity Index (5.4; 3.7; *p *= .033), relapse infections with the same microbe (22.2%; 1.1%; *p *= .001), and early-onset infections (< 30 days) (11.1%; 1.1%; *p *= .046), than the comparison two-stage exchange group without fractures. In contrast, age (72.5 study group; 68.2 comparison group; *p *= .224), rate of revisions for PJI in the past (55.5%; 51.7%; *p *= .827), and total re-infection rate (22.2%; 10.3%; *p *= .287) did not show a difference.

**Conclusion:**

PJI with co-existing cerclages for fractures were associated with multi-resistant microbes, relapse by the same microbe and early-onset re-infections. Cerclages might be considered a potential source of re-infection during a two-stage exchange. However, statistical weaknesses and a small study group must be considered limitations of the study.

## Introduction

A periprosthetic joint infection (PJI) is one of the most devastating complications of arthroplasty. Due to an aging society and an increasing acceptance for arthroplasties in younger patients, both the absolute number of arthroplasties and number of PJI has increased in recent years [1, 2]. PJI with contemporaneous fractures was described as “the worst-case scenario” by Müller et al. in a case series of eight patients, given the necessity to eradicate an infection on the one hand, and to stabilize a fracture using osteosynthesis material, with subsequent increased infection risk, on the other hand [3]. Existing clinical studies were able to show that arthroplasties implanted due to a fracture, for example, following a femoral neck fracture, have a higher risk of developing a PJI compared to the ones implanted because of a degenerative joint  disorder [4, 5]. Besides, fractures—among other causes—are a known general risk factor for development of a new infection after joint arthroplasty [6, 7]. However, up to this point, there are limited studies available concerning contemporaneous PJI and fractures.

This study analyzes a two-stage exchange protocol for patients with a PJI and a contemporaneously co-existing fracture treated with cerclages. As opposed to prior case series and reports, this study will compare outcome and characteristics of the patient cohort with patients treated with the same two-stage protocol and in the same department, but without co-existing fractures and cerclage treatment.

## Materials and methods

### Study design

The data were collected retrospectively out of a digital data bank, while all patients included in the study were followed up prospectively using a standardized protocol. The study was approved by the university’s ethics committee, including human and animal rights.

### Inclusion criteria

Criteria for inclusion were: (1) confirmed diagnosis of PJI treated with a two-stage exchange protocol, (2) a contemporaneous periprosthetic fracture treated with cerclage, (3) involvement of hip or knee joint, (4) and treatment from 06/2013 to 02/2016 in our department. Patients with (1) different PJI treatment strategies (one-stage exchange, debridement and implant retention), (2) fracture treatments other than cerclage (plates, screws), and (3) cerclages for extended trochanteric osteotomy (ETO) were excluded.

### Follow-up

Our departments follow-up schedule is based on follow-ups every 12 weeks within the first year, and then every 6 months. Analyzed parameters at the time of surgery included fracture classification according to AO [8]**,** microbe spectrum, patient age, age-adjusted CCI (Charlson Comorbidity Index) [9], and prior revisions. Follow-up parameters included re-infection and total re-revision rate during a 2-year follow-up.

### Treatment protocol

Every patient was treated with a long interval two-stage exchange for PJI [10]. The standardized protocol included a spacer less Girdlestone resection arthroplasty for all patients, followed by 2 weeks of i.v. antibiotics (AB). After 4 further weeks of oral AB, and if no signs of local or systemic infection were present, the prosthesis was reimplanted. Following the reimplantation, i.v. biofilm-active AB were administered for 1 week, and the protocol continued with 5 weeks of oral biofilm-active antibiotics, until a total of 12 weeks of continuously AB was completed. Initial calculated AB consisted of ampicillin/sulbactam (3 × 3 g i.v.) and additional vancomycin (2 × 1 g i.v.) in septic cases, MRSA-carriers, multiple prior surgeries, and suspected low-grade infection. As soon as a microbe was detected, targeted therapy was started, as proposed by Izakovicova and Trampuz et al. [11].

Germ detection was based on a standardized diagnostical algorithm. During the operation, at least four tissue samples were obtained. In addition, all foreign materials were sent in for sonication. The microbiological results summarized represent microbes identified through the total of all diagnostical tools.

### Definitions

A PJI was defined based on the European Bone and Joint Infection Society (EBJIS) [12]: (1) fistula or purulence around prosthesis, (2) leukocyte count in synovial fluid > 2000/μl or > 70% granulocytes (polymorphonuclear leukocytes), (3) periprosthetic tissue histology Krenn and Morawietz type 2 or type 3 [13], (4) microbial growth in synovial fluid or ≥ two tissue samples or sonication fluid. Re-infection was considered an absence of success following the Delphi-consensus criteria [14]: (1) wound healing without fistula, drainage, or pain, (2) no subsequent surgical intervention for infection after reimplantation surgery, (3) no PJI-related death (sepsis, necrotizing fasciitis). In addition, all re-infections were differentiated into new infections with involvement of a new microbe and relapses caused by the priorly identified microbe (Zmistowski and Parvici et al.) [15]. A symptom onset less than 30 days since the last surgery was considered an early infection [11]. Difficult to treat organisms (DTT) were defined as resistant to biofilm-active antimicrobials, including Rifampin-resistant staphylococci, Ciprofloxacin-resistant Gram-negative bacteria, and *Candida* [16].

### Statistics

Statistical evaluation was performed using SPSS (SPSS Inc., Chicago, IL, USA). Significance was considered *p *< 0.05. Significance tests for independent groups were used for calculation, including the *t* test and Mann–Whitney *U* test for continuous, and the Chi-square test for categorical variables.

## Results

Nine patients treated with both two-stage exchange for PJI and cerclage for fractures could be included. In two patients, two different fracture localizations were present. Thus, a total of 11 fractures were identified in nine patients. In one patient, cerclage implantation was performed before the prosthesis explantation, in six patients cerclages were implanted in the course of the prosthesis explantation, and in two cases, cerclages were implanted in the course of the prosthesis reimplantation. Fractures were all located in the femur (AO 3) with 2A3 as leading fracture type in five cases (2 Diaphyseal segment, A Simple, 3 Transverse < 30°), followed by 1A3 in three cases (1 Proximal end segment, A Trochanteric region, 3 Intertrochanteric), 3A2 in two cases (3 Distal end segment, A Extraarticular, 2 Simple), and 2B2 in one case (2 Diaphyseal segment, B Wedge, 2 Intact wedge) (Table 1).

**Table 1:** Fracture characteristics

Patients with cerclage implantation before prosthesis explanation	1
AO classification of treated fracture	3A2
Average number of cerclages	1
Patients with cerclage implantation during prosthesis explanation	6
AO classification of treated fractures	2A3 × 3
	1A3 × 3
	2B2
	3A2
Average number of cerclages	1.83
Patients with cerclage implantation during reimplantation	2
AO classification of treated fractures	2A3 × 2
Average number of cerclages	2

9.3% of all two-stage exchanges had a fracture as an additional complication (9 of 96 patients) (Table 2). No significant differences between two-stage exchange cases with (*n* = 9) and without (*n* = 87) an additional fracture could be identified concerning age (72.5 years; 68.2; *p *= 0.224), previous revisions for PJI (55.5%; 51.7%; *p *= 0.827), duration of hospital stay after surgery (33.8 days; 29.6; *p *= 0.389), total re-infection rate (22.2%; 10.3%; *p *= 0.287), and total re-revision rate (44.4%; 26.4%; *p *= 0.253) in a 2-year follow-up. In contrast, two-stage exchanges with additional fractures showed a significantly higher CCI (5.4; 3.7; *p *= 0.033), rate of early-onset infection (11.1%; 1.1%; *p *= 0.046) and relapse infections with the same microbe (22.2%; 1.1%; *p *= 0.001) compared to non-fracture cases. In addition, they showed a trend towards a later reimplantation after explantation (83.4 days; 64.9 days; *p *= 0.099).

**Table 2:** Two-stage exchange with and without additional fractures

Two-stage exchanges	Fracture/cerclage group	Comparison group	*p* value
Patients (*n*)	9	87	–
Knee/hip, (*n*)	3/6	44 / 43	.325^c^
Males/females, (*n*)	3/6	35 / 52	.687^c^
Age^a^	72.5 ± 5.0	68.2 ± 10.3	.224^d^
Age adjusted CCI^1^	5.4 ± 1.9	3.7 ± 2.1	**.033**^**e**^
Previous septic revision rate, (*n*)	5	45	.827^c^
Duration of hospital stay after surgery (days)^a,b^	33.8 ± 5.9 (25–44)	29.6 ± 14.2 (10–115)	.389^d^
Time between ex- and reimplantation (days)^a^	83.4 ± 50.4 (23–174)	64.9 ± 28.6 (7–198)	.099^d^
2-year follow-up			
Total re-infection rate, (*n*)	2	9	.287^c^
Early-onset re-infection (< 30 days after final treatment), (*n*)	1	1	**.046**^**c**^
Relapse, (*n*)	2	1	**.001**^c^
New-infection, (*n*)	0	8	.342^c^
Total re-revision rate, (*n*)	4	23	.253^c^

A* p* value < 0.05 was considered significant and printed in bold

^a^Mean ± SD (range)

^b^Duration of stay in days after explanation and reimplantation was added to a common sum

^c^Chi-square test

^d^*t* test

^e^Mann–Whitney *U* test

Coagulase-negative *Staphylococci* and *Cutibacterium* spp. were the leading microbes identified in both study and comparison group (Table 3). Patients treated with additional cerclages for fracture in the course of the two-stage exchange demonstrated a significantly higher rate of DTT microbes (44.4%; 8.0%; *p *= 0.001) and Gram-negative rods (*p *= 0.001). In both groups, high rates of polymicrobial cases were identified (47.1% and 66.6%).

**Table 3:** Microbe spectrum

Two-stage exchanges	Comparison group	Fracture group	*p* value^a^
Patients (*n*)	87	9	–
Coagulase-negative *Staphylococci* (*n*)	47	5	.930
*Cutibacterium* spp. (*n*)	17	3	.332
*Staphylococcus aureus* (*n*)	16	2	.779
*Enterococcu*s spp. (*n*)	9	2	.287
*Streptococcus* spp. (*n*)	8	1	.851
Gram-negative rods (*n*)	4	2	**.038**
*Candida* spp. (*n*)	1	2	**.001**
Further/unclassified (*n*)	15	3	.239
DTT (*n*)	7	4	**.001**
Monomicrobial (*n*)	41	2	.153
Polymicrobial (≥ 2 microbes) (*n*)	40	6	.237
Culture negative (*n*)	6	1	.643

A* p* value < 0.05 was considered significant and printed in bold

^a^Chi-square test

Cerclage material was introduced in the course of the second stage/reimplantation in two cases, thus not allowing for a sonication of cerclage material. In the remaining seven cases, cerclage material was exchanged in five patients (in one case together with the old prosthesis in the first stage, in the remaining cases, cerclages were introduced in the course of the first stage and exchanged or removed in the second procedure), and sent in for sonication. Microbe detection out of the five sonication samples was possible only in the case in which the cerclage was already present prior to the first stage, and also exchanged in the first stage. Two re-infections were noted in the cerclage group. In one case, the cerclage was removed in the course of the prosthesis reimplantation, and thus no cerclage could be sent in for sonication in the course of the re-infection diagnosis. In the second case, cerclage material could be analyzed for microbes via sonication, and identified *Staphylococcus aureus* as re-infection microbe, thus resembling the originally identified microbe spectrum.

## Discussion

PJI with a co-existing fracture is an important and challenging event. In the present study, nearly one in ten patients treated with a two-stage protocol for PJI had an additional fracture treated with a cerclage at the time of diagnosis or during the treatment itself. Exact data concerning prevalence of PJI and co-existing fractures are unknown, and can only be deducted from aseptic cases, where the rate of fractures after prosthetic hip revision is 4% [17], the one after knee prosthesis revision up to 30% [18]. Although exact demographics are undetermined yet, osteoporosis and metastasis in elderly patients on the one, and more active and mobile patients on the other hand, will likely also increase the number of fractures in combination with PJI in the nearby future.

The most important fracture characteristics in our study included a strong preference towards the hip joint and femur, as well as intraoperatively caused fractures. The femur as leading fracture location is typical for periprosthetic fractures in both hip and knee [19]. Besides, several described general risk factors for fractures are present in our patients such as an age older 70 years, prosthesis loosening and a poor overall patient condition [20, 21].

The present study was able to show that a combined therapy using a two-stage exchange and cerclages can efficiently treat PJI with co-existing fractures, resulting in no significantly (*p *= 0.287) higher total re-infection rates compared to non-fracture PJI in the course of a 2-year follow-up. However, fracture/cerclage cases demonstrated a significantly higher rate of early-onset infections and relapses by the same microbe. Janz et al. were able to show that cerclages after femoral osteotomy are a risk factor for bacterial colonization during a two-stage septic total hip arthroplasty. This might explain the higher rates of early-onset and relapse infections found in this study. However, Janz et al.’s study did not include patients with fractures but iatrogenic caused femoral osteotomies (stem mobilization). Besides, the study did not have a standardized comparison group without osteotomies and cerclages [22].

In general, the high rates of *Staph. epidermidis* (coagulase negative) and *Staph. aureus* identified  in our study are similar to the rates described in most PJI studies without additional fractures [1, 3, 4, 23]. However, compared to prior studies, high rates of DTT such as *Candida* were identified in the present study. Usually, *Candida* as a PJI causing microbe is involved in less than 1% of all PJI [1]. This study is the first one to find a significantly higher DTT rate in two-stage exchanges with fractures treated with cerclage compared to cases without additional fractures (*p *= 0.001). Up to this point, no literature equivalent describing this phenomenon is present. Of the four DTT cases in our study that underwent two-stage revision, one had a preoperative existing fracture, while the others were caused intraoperatively iatrogenic during stem explantation, and in two cases, in the course of the reimplantation. Preoperative existing fractures could facilitate DTT attachment, or DTT cases could react osteolytic/structure damaging, contributing to more intraoperative fractures. In addition, present cerclages might allow for a possible biofilm formation and attachment in the joint. However, the exact relation between DTT and fractures remains undetermined. Age and revision rate for PJI in the past did not seem to influence this correlation. Instead, the small number of patients and a general tendency towards sicker patients (CCI 5.4; 3.7; *p *= 0.033) should be considered as additional explanations. To allow for an exact statistical calculation, a matched pair analysis would be necessary. However, given the overall number of only nine cases in the study group compared to 87 patients in the control group, this analysis itself would be of limited evidence. Further research on a larger patient scale might be necessary in the future.

Despite being the first study to systematically describe characteristics and outcomes of PJI with co-existing factures, structural problems and remaining questions should be critically discussed. A number of questions cannot be answered using the current methods.

As the statistical outcome remains inconclusive, it remains undetermined, if periprosthetic fractures are associated with a poorer outcome. In this context, it also remains unknown if cerclages as biofilm colonized foreign body are the main cause of a persistent infection, or higher rates of relapse infection by the same microbe and fractures are confounding bias of an overall sicker population. Sonication of cerclages might seem like an option to answer this question. However, while sonication of cerclage material might be useful in the course of the first stage (exchange of prosthesis), and thus before the start of antibiosis, the sonication of cerclages removed/exchanged in the second stage is problematic, as prior antibiosis might cause false-negative results. To give a resume, limitations must be addressed before drawing final conclusions, such as the necessity to exchange cerclage, introduced during prosthesis removal, in the course of the reimplantation of a two-stage exchange.

## Conclusion

Fractures treated with cerclage are an additional complication with a prevalence of about 10% in two-stage revisions for PJI. The diaphyseal region of the hip was the most prevalent fracture localization, with most fractures being caused intraoperatively iatrogenic. *Staph. epidermidis*, *Cutibacterium* and *Staphylococcus aureus* were the most prevalently identified microbes. Contemporaneously fractures treated with cerclages in the course of a two-stage exchange showed a higher rate of difficult to treat microbes, relapse infections by the same pathogen and early-onset re-infections than patients without additional fractures. Nonetheless, limitations, including limited absolute patient numbers and subsequent statistical weaknesses, must be addressed before drawing final conclusions.

## Data Availability

Made available upon request.
